# Ethnobotanical Knowledge in Sete Cidades, Azores Archipelago: First Ethnomedicinal Report

**DOI:** 10.3390/plants8080256

**Published:** 2019-07-30

**Authors:** Pedro T. M. Silva, Marta A. F. Silva, Luís Silva, Ana M. L. Seca

**Affiliations:** 1 Faculty of Sciences and Technology, University of Azores, Rua Mãe de Deus, 9501-801 Ponta Delgada, Portugal; 2 InBIO, Research Network in Biodiversity and Evolutionary Biology, CIBIO-Açores, University of the Azores, 9501-801 Ponta Delgada, Portugal; 3 cE3c-Centre for Ecology, Evolution and Environmental Changes/Azorean Biodiversity Group & University of Azores, Rua Mãe de Deus, 9501-801 Ponta Delgada, Portugal; 4 QOPNA & LAQV-REQUIMTE, University of Aveiro, 3810-193 Aveiro, Portugal

**Keywords:** ethnobotany, ethnomedicine, medicinal plants, plant diversity, use value, *Clinopodium menthifolium* subsp. *ascendens*, *Morella faya*, Azores

## Abstract

Knowledge about folk medicines is limited to elder community members of remote communities, like Sete Cidades in the Azores. The Azores, 1300 km west of Portugal, are nine volcanic islands, totalling 2330 km^2^ of land dispersed by 173,200 km^2^ in the North Atlantic Ocean. The present study aims to scientifically document the uses of plant species for medicinal purposes, in the Sete Cidades. Twenty-eight community members from 40 to 84 years of age, of whom half were 55 to 64 years old, were interviewed. Twenty-nine taxa were reported as being used for medicinal purposes, ten of which have not been previously reported for ethnomedicinal use in Portugal, with a first record of the use of *Morella faya*. Leaves were the most used plant part (55%), and decoction the most common preparation mode. The five reported taxa with both the highest use value (0.71–0.25) and relative frequency of citation (0.14–0.11) were *Clinopodium menthifolium* subsp. *ascendens*, *Aloysia citriodora*, *Mentha x piperita*, *Citrus limon* and *Rosmarinus officinalis*. The traditional uses of some of the reported plants are supported by scientific studies, confirming their ethnomedicinal value and the need to preserve local knowledge of folk medicine practices.

## 1. Introduction

Knowledge about the medicinal properties of plants has been transmitted from generation to generation, and is directly linked to the local culture, faith and other beliefs, and is now the study object of ethnobotany and ethnopharmacology [1,2]. Nowadays, in rural societies and developing countries, the use of medicinal plants is both a necessity and valuable resource, providing an alternative for primary health care systems, income generation and livelihood improvement, while in developed countries, traditional medicine is used as a complementary therapy, and the demand for it is increasing [3,4,5]. However, this cultural knowledge is under threat of extinction because it is limited to a small portion of society, mostly residents in remote regions, and because of the degradation of the local ecosystems [6]. Thus, the assessment of the traditional knowledge of plant uses through ethnopharmacological surveys, and its documentation, is essential for the conservation of the local culture and of natural resources, being also an incentive to research the use of plants with a view to providing scientific substantiation of reported medicinal applications. The Azores Archipelago (Portugal) is one such remote regions where, although already under risk, there are still aged communities in isolated rural areas, for whom the use of folk medicines is very relevant, but where systematization of the associated ethnopharmacological information has not been undertaken.

**Figure 1:**
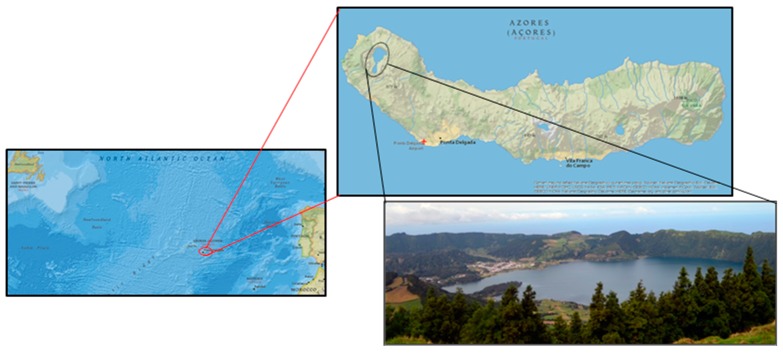
Photo of Sete Cidades village, its position on the map of São Miguel Island, Azores, and the relative position of Azores archipelago in the Atlantic Ocean (Maps are not to scale).

The archipelago of the Azores is composed of nine small islands located in the North Atlantic Ocean (Figure 1), nearly 2330 km^2^ of land dispersed along 557 km in the east-west direction, and 311 km in the north-south direction, at about 1370 km west of Portugal and 4100 km east of New York [7].

Many Azorean villages are located in isolated geographic areas, often inside volcanic craters, with populations dedicated to subsistence agriculture, and where the knowledge and tradition of using medicinal plants carry on [8,9,10]. The Azorean vascular plant flora that includes approximately 1000 vascular plant taxa, with about 200 to 300 native species, approximately 700 naturalized, and many more introduced along the 500 years of human presence [11], provides the plant resources used as folk medicine in the region. In the last two decades, accessibility to some of these villages has improved, allowing easier access to modern health care and bringing about changes in the demographic, social and cultural structure of the most isolated populations [12]. One of these changes is the higher probability of abandonment or adulteration of the traditions and, with this, the loss of ancient knowledge, such as medicinal plant uses for primary health care, as happened in other regions around the world, (e.g., [6,13,14]). Informal records of medicinal plant use exist in the Azores [8,9,10]. However, no scientific ethnobotanical studies have been conducted reporting primary data for a geographically-isolated rural community. On the other hand, since the settlement of the region originated from the mainland Portuguese population [15,16], it will be relevant to evaluate the similarities and divergences in the use of medicinal plants in both regions.

The present study, using proper plant use documentation, unambiguously linked to specimens and qualitative and quantitative analyses of the collected data, aims to: i) describe and document the uses of plant species for medicinal purposes, in the small and relatively isolated community of Sete Cidades in São Miguel Island, Azores; ii) based on the pharmacological effects and chemical profile described in the literature for the most relevant species, assess the scientific basis for the traditional applications described; and iii) promote the preservation of folk knowledge and local flora, clarifying knowledge about its medicinal use.

## 2. Results

### 2.1. Informant Characterization

**Figure 2:**
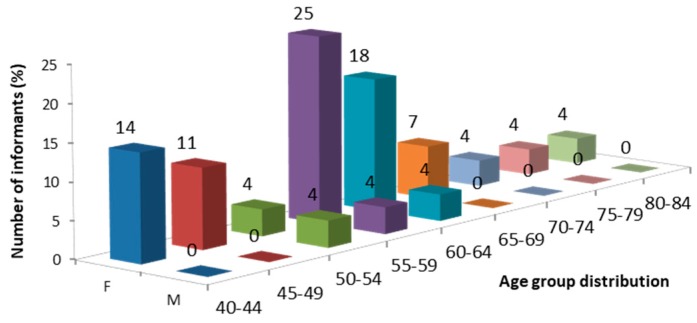
Age and gender group distribution of the informants (F-female; M- male).

Knowledge about the traditional uses of medicinal plants was found mostly among female members of the community (89.3 % of the interviewed individuals were female), among whom 50% were between 55 and 64 years old (Figure 2).

When analysing the source of this “medicinal knowledge”, the majority of informants (78.5%) stated that it came exclusively from family tradition, that is, knowledge transmitted by ancestors. Moreover, many of the informants mentioned that younger people were not interested in learning folk medicine.

### 2.2. Ethnomedicinal Flora

In the present work, the informants mentioned a total of 29 plant taxa as utilized in traditional remedies for the treatment of a broad range of human ailments (Table 1). These plant species belong to 16 families, the Lamiaceae being the most represented with eight species. It was found that 20.7 and 27.6% of the reported taxa were indicated for one and two types of ailments, respectively, while 51.7% of all reported taxa were indicated for the treatment of three to seven different ailments. These results indicate the great versatility of most plants used as traditional herbal remedies by this population.

Additional information collected during interviews concerning the method of preparation, posology and possible adverse effects of each plant is reported in Appendix A (Table A1).

Due to the fertile soils and favourable climatic conditions, an abundance of the species reported in Table 1 throughout the year is observed. Accordingly, the local inhabitants, in most cases, do not have to resort to plant conservation (by drying or other methodology), since they can easily get them fresh.

### 2.3. Target Diseases or Conditions

Most of the plants reported in Table 1 have been used by the Sete Cidades community to treat diseases that affect the gastrointestinal system (18 different taxa, 62.1%), such as stomach pain (12 taxa), diarrhoea (7 taxa) and indigestion (3 taxa). This community uses 7 taxa (24.1% of the all taxa) to treat respiratory system diseases like colds, cough and bronchitis, while they use 6 different taxa for the treatment of abdominal cramps whose aetiology was very vaguely defined by the informants; to treat “nerve problems” or urinary problems, such as kidney and bladder pains, they use 5 different taxa each. Some of the reported plants exhibit a particular role in the well-being of this community, such as *Clinopodium menthifolium* subsp. *ascendens* (Jord.) Govaerts, *Morella faya* (Aiton) Wilbur, *Petroselinum crispum* (Mill.) Fuss and *Taraxacum campylodes* G.E.Haglund, each being the only one used as an insect repellent, to fortify the hair, to improve de memory and as a laxative, respectively.

### 2.4. Administration Routes

The present study registered four different routes of administration of the herbal remedies, with ingestion being the most used, followed by dermal application, inhalation; the least used was gargle. Among the administrations by ingestion, water infusion and decoction of leaves, flowers and peels were the most reported forms of preparation in the study area, while juices, salads and soups were rarely reported. Regarding dermal applications, the maceration of flowers and leaves, and the decoction of leaves were the most common uses. Most of the dermal uses are by direct application on the skin, while others are applied in bath water (Table 1).

In some cases, decoction, infusion and juice preparation do not involve a single plant, but rather, a mixture to increase efficiency.

### 2.5. Plant Parts Used in the Present Study

**Figure 3:**
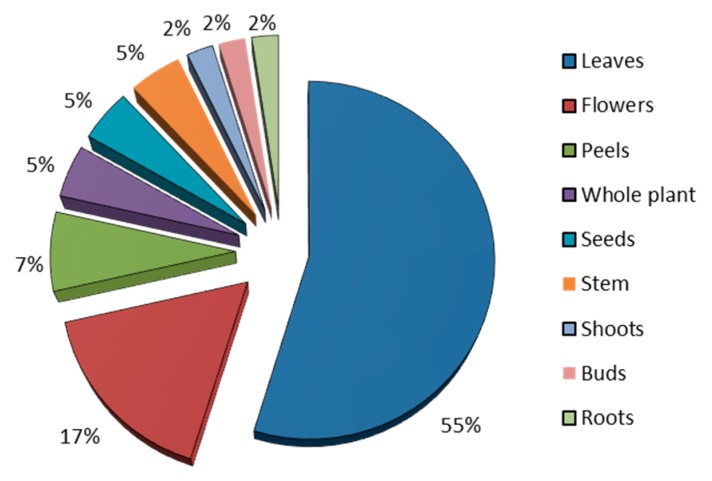
Use-reports of plant parts for herbal preparation in the Sete Cidades community.

The plant parts used for medicinal purposes by the informants are reported in Table 1 and graphically represented in Figure 3. Leaves were the most used part (23 of use-reports, 55%), followed by flowers (7 of use-reports, 17%), while the other plant parts are cited for less than four use-reports each.

Although for most plants reported in this study, only one part of the plant is used, there are some cases where several parts of the same plant are used, either mixed in the same preparation (e.g. maceration of leaves and flowers of *Rosmarinus officinalis* to treat blood circulation problems), or separately for the treatment of different disorders (e.g. *Morella faya*, whose burnt seeds are used in wound care while a decoction of the leaves is used to fortify the hair and to treat abdominal cramps and leg pain).

### 2.6. Use Value and Comparative Analysis of Medicinal Plants Importance

In the present study, as presented in Table 1, the UV ranged from 0.04 (i.e. *Chelidonium majus, Dysphania ambrosioides*, *Elaeagnus umbellata*, *Equisetum telmateia*, *Fragaria vesca*, *Hedera helix*, *Origanum majorana*, *Thymus vulgaris* and *Tilia × europaea*), to 0.71, in which the highest UV was recorded for *Clinopodium menthifolium* subsp. *ascendens*, followed by *Aloysia citriodora* (0.50), *Mentha x piperita* (0.29), *Citrus limon* (0.25), *Foeniculum vulgare* (0.25) and *Rosmarinus officinalis* (0.25). Bearing in mind that the higher the UV value, the higher the relevance of this plant to the community [17], these high use values show how these taxa are relevant to the local community and are commonly used in local folk medicine. The remaining species (14) were reported to have UV values between 0.07 to 0.18. However, as referred by Dudney et al. [18], the UV parameter cannot distinguish the number of informants who cite the species or consensus among those uses, so analyses of the species’ importance deduced only from the UV value are quite limited; accordingly, the use of other parameters, such as the RFC, is recommended.

The highest RFC value was recorded for *Clinopodium menthifolium* subsp. *ascendens* (0.43), also with the highest UV, which shows how relevant this species is to the local community. In fact, twelve informants (43%) mentioned its use in local folk medicine, and each indicated, on average, 1–2 uses (UV/RCF = 1.6). The other five taxa with the highest RFCs were *Aloysia citriodora* (0.14), *Citrus limon* (0.11), *Malva sylvestris* (0.11), *Mentha x piperita* (0.11) and *Rosmarinus officinalis* (0.11). It should be noted that five taxa showed both the highest UV and RFC values (*Clinopodium menthifolium* subsp. *ascendens, Aloysia citriodora*, *Mentha x piperita*, *Citrus limon* and *Rosmarinus officinalis*).

The local people use the species highlighted in the above paragraphs for the treatment of many diseases. As can be seen in Table 1, ten different uses were given for *Clinopodium menthifolium* subsp. *Ascendens*, which means a potentially high versatility but a low degree of use consensus for this taxon. Additionally, the remaining four taxa with the highest UV and RFC values were reported for the treatment of 9, 6, 4 and 4 different ailments, respectively.

The results presented in Table 1 also reported the medicinal uses of *Morella faya*, albeit with low UV and RFC values (UV = 0.14; RFC = 0.07). The fruits of this species are edible and are used in the preparation of liqueurs and jams, but no medicinal applications were found for this taxon from any previous ethnomedicinal studies on the Scopus and Web of Science databases.

## 3. Discussion

### 3.1. Discussion of Informant Profile, Target Diseases and Administration Routes

The informant profile (Figure 2) highlights the relevance of the women as guardians of traditional knowledge of the use of plants for primary health care. Presumably, because in these isolated communities, even today, within the family, women are in charge of taking care of the sick, and as such, are the "keepers" of knowledge about medicinal plants [19]. The lack of interest among young people for learning this traditional knowledge shows a significant increase in the risk of its loss. Probably, this is because younger people have less movement restraints, and so have more access to modern healthcare facilities, trusting more in modern medicine [20,21].

The ailment with the most indications, as reported by the informants, was gastrointestinal disorders, which agrees with the data recorded in other studies in Portugal [22,23,24], Spain [25,26] and around the world [27,28,29,30]. Presumably, this is mostly due to unsatisfactory dietary conditions and food hygiene deficiencies [31].

Reported routes of administration match with data from other studies [23,28], except for gargling, which was described by Neves et al. [23] as also being frequent. Studies in other areas of Portugal [22,23] also reported water infusion and decoction of leaves, flowers and peels as the most commonly reported forms of preparation. The population of the community of Sete Cidades descends from families originating from mainland Portugal who arrived at São Miguel Island [15]; this is a possible reason for the similarities in the modes of administration and preparation.

The present study reports the use of plant mixtures for the most effective treatment of diseases. This practice has also been reported in other studies [32,33,34]; perhaps its efficacy results from a synergistic effect between the compounds of different plants.

Different parts of the plant exhibit distinct profiles of compounds, and therefore, exhibit different biological activities, and are used as traditional remedies to treat several diseases [35,36]. As shown in Figure 3, the leaves are the most used part of the plant. According to Bonet et al. [37], this fact, also reported in other studies, is due to the abundance, as well as the ease of harvesting and renewal of leaves.

### 3.2. Scientific Evidence to Support Ethnomedicinal Report

Research in the Web of Sciences and Scopus databases allowed us to report here, for the first time, the ethnomedicinal use of *Morella faya*. However, the non-English document "Inventario Español de los conocimentos tradicionales relativos a la biodiversidad" indicates several medicinal uses of this plant in the Canary Islands—mainly its bark and aerial parts—for the treatment of ailments such as diarrhoea, cough, wounds, flu and as an ingredient in a mixture used to treat fistulas [38]. The medicinal applications of *Morella faya* in Sete Cidades community have much in common with those described above, i.e., the healing and analgesic effects.

*Morella faya* leaves and berries extracts exhibit antioxidant effects and inhibit some digestive enzymes; their major chemical constituents are polyphenolic compounds such as galloyl esters of flavonoids and phenolic acids, i.e., compounds that yield positive effects on human health [39,40]. Given that wounds and pains are often associated with inflammatory processes, and that the antioxidant action is beneficial in reducing inflammation [41], it is reasonable to assume that the antioxidant effect referred to above supports the medicinal application of *Morella faya* as an analgesic.

A literature review showed that several uses reported in the present study are not exclusive to the Sete Cidades community, which confers a certain degree of use validation, although without scientific character. In fact, according to Çakılcıoğlu et al. [42], the pharmacological effect of a specific plant could be accepted and explored if the same plant is used in different parts of the world to treat the same disease. Nearly 50% of the species reported in Table 1 have at least one use in common with those described in the literature. Regarding only the most important species in this study (UV > 0.25 and RFC > 0.11), the most similar uses are discussed below.

On the island of Madeira, the leaves of *Clinopodium menthifolium* subsp. *ascendens* (syn. *Clinopodium ascendens* (Jordan) Sampaio), are used to treat toothache [43]. However, this information does not come from a scientific ethnomedicinal report. Additionally, in north-eastern Catalonia, a tisane of *Clinopodium menthifolium* subsp. *ascendens* and *Antennaria dioica* (L.) Gaertn. mixture is used as a tranquillizer [34]. An infusion of *Aloysia citriodora* leaves is used in another community of São Miguel Island to treat stomach pain and psychic tension [9], and as a calmative and sleeping aid in the Northern Badia region, Jordan [44]. An infusion of *Mentha x piperita* leaves is used in mainland Portugal as an antihelmintic for children, and to treat stomach pain and flu [22], and as digestive [23], while a decoction of the *Citrus limon* fruit peels is used to treat rheumatism [9], colds [22,45,46] and flu [46]. Also, in Portugal, an infusion of *Rosmarinus officinalis* aerial parts is used to treat headaches and blood circulation problems [22], while stomach pain is treated with an infusion of the plant’s leaves and flowers [23].

In order to guarantee the medicinal value of plants, more attention should be given to scientific studies that could support their use.

The antibacterial, anti-inflammatory and analgesic effects of *Chelidonium majus* described in the literature [47,48] could support the topical used of latex or juice to treat scratches and wounds, as reported in the present study (Table 1). The folk use of *Dysphania ambrosioides* (syn. *Chenopodium ambrosioides* L.) to kill intestinal worms (Table 1) can be justified by the presence of ascaridole and unidentified hydrophilic components which exhibit anthelmintic activity [49,50].

In contrast, although the aerial parts of *Ruta chalepensis* exhibit several biological activities [51,52,53], an in vivo study relating to the analgesic activity of this plant showed inactivity of the ethanolic extract [52]. Thus, the use of an infusion of the plant’s leaves by the Sete Cidades community as an analgesic, to treat kidney, bladder, stomach and spine pains (Table 1), is not supported by scientific studies. However, it is possible to feel some relief from these ailments if an inflammatory process is involved, since *Ruta chalepensis* extract exhibited an anti-inflammatory effect in vivo and in vitro [52,54]. Also, the use of *Sambucus nigra* by the Sete Cidades community as an antidiarrheal and antipyretic (Table 1) does not seem to be supported by scientific studies. In fact, despite the broad biological activities of *Sambucus nigra* [55,56,57,58], the consumption of the immature plant or high quantities of fruits may cause diarrhoea itself, as well as nausea and vomiting [59].

Scientific studies of *Clinopodium menthifolium* subsp. *ascendens*, the species with the highest UV and RCF values (Table 1), are often published under a synonymous name such as *Calamintha ascendens* Jord*, Calamintha sylvatica* subsp. *ascendens* (Jord.) P.W.Ball, *Calamintha officinalis* subsp. *ascendens* (Jord.) Mateo or *Clinopodium ascendens* (Jord.) Samp., among others [60], and these names are not always correctly written. Taxonomic reclassifications, derived from the difficulty of distinguishing among closely related taxa, make the tracking of studies challenging and sometimes confusing. To our knowledge, there are no studies on the chemical composition or biological activities of *Clinopodium menthifolium* subsp. *ascendens* leaves or aerial parts (besides the properties of its essential oils [43,61,62,63]) that could support the plant’s use given by the Sete Cidades community.

### 3.3. Toxicological Evidence of Medicinal Plants

In order to ensure the safe use of the medicinal plants reported in Table 1, the toxicity of the species used by the community of Sete Cidades was evaluated, taking into account the informants’ information and the published literature.

For thirteen of the twenty-nine plants listed in Table 1, informants indicated that the preparation “... should not be given to children under five years old” (Table A1 in Appendix A). The justification for this is very general, i.e., it “... makes these children feel bad”.

For ten of these species, *Allium cepa*, *Allium sativum*, *Aloysia citriodora*, *Elaeagnus umbellata*, *Malva sylvestris*, *Melissa officinalis*, *Morella faya*, *Petroselinum crispum*, *Psidium cattleianum* and *Rubus ulmifolius*, there are no scientific studies to support the informants’ warnings. In fact, to the best of our knowledge, these plants are not considered toxic [64,65], and there are no published studies on their toxicity in children. Perhaps this warning is more related to the preventive and protective social conscience that considers children under five years old as very fragile. Despite the apparent non-toxicity of these plants, the use or consumption of medicinal plants should never exceed the limits recommended by WHO [3].

However, for three species, *Ruta chalepensis*, *Mentha x piperita* and *Sambucus nigra*, the informants’ warning seems to make sense, since some adverse effects, at least for vulnerable groups such as children and pregnant women, are described in the literature and are discussed below.

For *Ruta chalepensis*, in addition to the warning about the toxic effects on children under five years of age, the informants also warn that an “infusion of more than 20 g of the leaves per litre gets toxic”. The literature describes the in vivo assay, showing that an infusion of the leaves in daily doses causes an embryotoxic effect [66], while an ethanol extract of aerial parts of the plant induces a significant depressant effect on the central nervous system [51].

Although *Mentha × piperita* is generally recognized as safe [67,68], it exhibits some adverse effects, mainly derived from the presence of pulegone and menthol metabolites, which are described as not recommended for young children [68,69].

Also, the species *Sambucus nigra* is not considered a toxic plant. However, its roots, bark, stems, leaves, flowers and unripe fruits are known to contain cyanogenic glycosides, mainly sambunigrin, prunasin, holocain and zierin, which are converted, during digestion, into toxic hydrogen cyanide [70]. Fortunately, and according to Ulbricht et al. [71], the heating process causes degradation of cyanogenic glycosides, reducing the risk of poisoning. Thus, the informants’ warning seems to have some scientific fundament, and is also in compliance with the European Food Safety Authority [72], which included this species in the “Compendium of botanicals reported to contain naturally occurring substances of possible concern for human health”. EMA also recommend avoiding the ingestion of *Sambucus nigra* fruits by pregnant and lactating women, as well as children and adolescents under 18 years old [73].

Despite the absence of indication by the informants for possible toxic properties, some plants used by the community of Sete Cidades are described in the literature as having, in some circumstances, adverse effects. This is the case of *Chelidonium majus*, for which the ingestion of its latex or the application of the plant directly on the skin cause severe irritation of the digestive mucosa and contact dermatitis, respectively [74]. There is also controversy regarding the hepatotoxic and hepatoprotective effects of *Chelidonium majus* [47,75,76]. So, special precautions should be taken with *Chelidonium majus* administration for people with liver problems, or during pregnancy and lactation [76].

In addition, it should be borne in mind that São Miguel Island still has active volcanism and that plants can absorb various types of nutrients and soil elements such as heavy metals. Therefore, an investigation should be carried out on the concentration of heavy metal concentrations in the plants of the Sete Cidades area, in order to ensure the safe use of herbal products.

Many questions remain unanswered regarding scientific support for the traditional use of the medicinal plants reported in Table 1, mainly due to a lack of studies on the biological activities (in vitro, in vivo) and toxicological effects of the compounds and extracts.

### 3.4. Comparative Analysis of the Reported Medicinal Flora with other Portuguese Regions, Iberian Peninsula and World

In the past, ethnomedicinal and ethnobotanical studies have involved a single culture or one ethnic group [77], while in the last few decades, more attention has been given to comparative studies concerning multiple communities, contributing to increasing the intercultural importance of medicinal flora among different ethnic groups across the globe [78]. This recent approach is also important for highlighting the significance of cross-cultural variations and opening new research prospects for medicinal plants [30].

The flora, climate and geographical position of the Azores are very different from those in mainland Portugal or other islands which are much closer to continental coasts such as Madeira, Porto Santo and the Canary Islands. However, taking into account the origin of the population that started the settlement of the Azores, it is relevant to compare the medicinal flora reported here with the ethnobotany and ethnomedicinal reports from other regions of Portugal [22,23,24,45,79] (Table 2 and Table A2 in Appendix A).

Analysing the data from Table 2 and Table A2 (Appendix A), a total of 19 species were previously reported in ethnobotanical studies in other Portuguese regions (about 65% of the total species reported in Table 1). *Foeniculum vulgare* was reported in all 5 studies, while *Chelidonium majus*, *Melissa officinalis*, *Mentha piperita*, *Rosmarinus officinalis* and *Sambucus nigra* were cited in 3 of the 5 studies. This figure, i.e., 65%, suggests the presence many medicinal plants which are common to different regions of Portugal, which indicates a high interregional consensus on which plants are of medicinal interest. The similarity of plants and uses between the Sete Cidades and other Portuguese communities, together with most informants identifying their ancestors as the source of the knowledge about medicinal plants, reinforces the idea that these ancestors may have a common geographical origin.

The ten species not reported in previous Portuguese studies are *Allium sativum*, *Aloysia citriodora*, *Clinopodium menthifolium* subsp. *ascendens*, *Dysphania ambrosioides*, *Elaeagnus umbellata*, *Mentha spicata*, *Origanum majorana*, *Taraxacum campylodes*, *Tilia × europaea* and *Morella faya*. Half of these unreported plants are being traditionally used as folk remedies in other regions of the Iberian Peninsula. For instance, the bulb of *Allium sativum*, the latex of *Taraxacum campylodes*, the aerial parts of *Mentha spicata* and the flowery plant of *Aloysia citriodora* are used in folk medicine in the Arribes Del Duero, Granada and Navarra regions [25,26,80], while the flowery aerial parts of *Origanum majorana* are used in Andalusia [81] to treat a variety of diseases.

Several of the plants reported in Table 1 have ethnomedicinal value in other parts of the world, since they were mentioned by different communities, albeit for the treatment of diseases other than those indicated in Table 1. *Allium cepa* is used in the Lakki Marwat District, Pakistan, to prevent heart diseases, as an antihypertensive agent, and against insect bite hypersensitivity reactions [28]. In Sardina, Italy, *Malva sylvestris* is used as a mild laxative as an anti-inflammatory and to treat toothache, stomatitis, dermatitis, menstrual pain, hypertension and weakness [82]. Tribess et al. [83] report the use of *Petroselinum crispum* in Southern Brazil to treat abdominal cramps, bladder/kidney problems and uterus inflammations. A powder of *Origanum majorana* is utilised by herbal practitioners of Bajaur Agency, Pakistan, to treat hypertension and general body pains [30]. Use of aerial parts and fruits of *Rubus ulmifolius* has been reported in the folk medicine of Montecorvino Rovella to treat acne [84].

### 3.5. Sustainable Harvesting, Commercialization and Cultivation of Medicinal Plants

For many years, in the study area, there has been a continuous harvest for personal use of the plants reported in Table 1. Many informants, i.e., 79%, reported having plants in their own or in a neighbouring garden or yard, and 82% indicated that they had plants available to use throughout the year. Thus, harvesting appears to be sustainable, as there is no evidence of biodiversity loss or overexploitation of any of the species reported in Table 1. However, on São Miguel Island, over the last 3-4 years, the interest in natural products and medicinal plants has been growing, and it is already possible to find the first marketing points for some of these plants. Thus, it seems that the commercial value of some plants is increasing, albeit slowly, thereby jeopardizing sustainability.

On the other hand, considering the abundance and ease of planting of some plants, the development and implementation of sustainable cultivation, harvesting and marketing for some of these medicinal plants could contribute to the creation of new value-added products. It could also be a way of contributing to the dissemination of knowledge about the use of these medicinal plants in local society, since at present, the risk of the disappearance of this popular knowledge has been declared to be greater than the risk of the loss of the natural resources themselves. [85].

## 4. Materials and Methods 

### 4.1. Study Area and Its Socioeconomic Background

The study area, Sete Cidades, is located at 37°87’N latitude and 25°78’W longitude, on the western part of the São Miguel Island, Azores Archipelago, in the North Atlantic Ocean (Figure 1). The Sete Cidades village is situated at 260 m altitude inside of a volcanic caldera with 5 km diameter and walls ranging from 400 m to 856 m in height. This caldera is dominated by a large lake (Lagoa das Sete Cidades), artificial pastures in the flatter areas around the lake, and with forested area dominated by *Cryptomeria japonica* (D.Don (Cupressaceae), *Pittosporum undulatum* Vent., (Pittosporaceae) and *Acacia melanoxylon* R.Br. ex W.T.Aiton (Fabaceae), and some fragments of the native vegetation at the highest and steepest points [86]. The volcanic caldera of Sete Cidades is classified as a protected landscape and is included in São Miguel Island Natural Park [87]. The climate is mesothermic, marked by oceanic characteristics, and the caldera gets an average of 1800 to 2200 mm of precipitation, reaching the maximum in the southeast flank, where it surpasses 2600 mm [88,89]. The population of Sete Cidades in 2011 comprised 793 inhabitants, having decreased by 7.6% in the previous ten years, mainly among the 0–24 age group (decrease of 28.6%) [90]. The study area has limited education facilities (one elementary school) and does not possess hospitals or modern health facilities. Due to all these constraints, the use of medicinal plants is a continuous practice in the study area. In the last decade, accessibility to the largest population centre has been improved, reducing the isolation of the Sete Cidades community, but also increasing the risk of the disappearance or adulteration of traditional practices concerning medicinal plants.

### 4.2. Ethnomedicinal Data Collection and Ethnographic Composition

The community of Sete Cidades was chosen because it is a relatively small and isolated rural community, limited in access to health services, with a small and aged population. These characteristics facilitate the preservation of empirical knowledge, increasing the likelihood of finding people with unadulterated knowledge about the use of plants for medicinal purposes.

Data collection was performed through interviews with the study area residents from February to July 2015 and July 2016, following the conceptual and methodological practices accepted in ethnopharmacological field studies [91,92]. The study was performed by Portuguese people in a Portuguese territory, with each informant verbally giving informed consent for data collection. The questionnaire consisted of 14 questions in Portuguese, mostly with semi-closed answers, allowing spontaneity in the interview and avoiding information loss. Twenty-eight informants were interviewed, 25 females and 3 males, from 40 to 84 years of age (Figure 2). Informants were asked for information about the plants in the study area used to treat human diseases, including vernacular names, time of harvest, parts of the plant to use, method of preparation, form and duration of treatment, disease/symptom to treat, etc. (Table 1 and Table A1 in Appendix A). Interviews were conducted individually at home and/or at the community meeting house (“Casa do Povo”), and focused on people considered by the community to have a broad knowledge of the subject.

### 4.3. Preservation and Taxonomic Verifications of Plant Species

The interviews were followed by field trips to collect and identify the target plants in the areas where informants usually collect them (home garden and yard and in the surrounding areas), with their assistance/presence; samples were then taxonomically identified by the Prof. Luís Silva from the Department of Biology, University of the Azores, following standard floras and international databases [11,93,94,95,96,97]. A voucher was prepared by standardized procedures and deposited at the registered Herbarium Rui Telles Palhinha (AZB), University of the Azores, where a voucher number was assigned (first column of Table 1). The names indicated in the tables in this work are the accepted names according to the “The plant List” database [60].

### 4.4. Data Analysis

Collected data were analysed using two different quantitative indices: use value (UV) and relative frequency of citation (RFC). 

The UV index was used to evidence the citation value of each plant (UVs) used by the inhabitants of Sete Cidades, and was calculated as follow:

UV_s_ = Σ*U_i_*/*N* (1)

where Σ*U_i_* is the sum of total number of uses by all of informants for a specific plant and *N* shows the total number of informants interviewed in the survey [17,98].

Ethnomedicinal data collected during the survey was analysed quantitatively by calculating the RFC index as follows:

RFC = *FC*/*N* (2)

where *FC* is the number of respondents who mentioned the use of the plant, and *N* displays the total number of informants interviewed in the survey [99]. RFC directly relies on the number of informants that reported a certain plant, indicating the local importance but without considering the ailments for which it is used [100].

## 5. Conclusions

The present study collected relevant information about the medicinal plants used by the community of Sete Cidades, helping to preserve local knowledge about the uses of plants in this region and to attract future generations to the use of folk medicines.

The study reported on the use of 29 plants in the treatment of treat gastrointestinal disorders and diseases of the respiratory system, among others. Nineteen of these species are also referred to as medicinal plants in ethnobotanical studies conducted in other regions of Portugal. Almost 50% of the 29 species reported in this study exhibit identical uses to those reported for the same plant and part of the plant in other regions, mainly in Europe and the Middle East.

After searching the Web of Sciences and Scopus databases, it appears that the ethnomedicinal use of *Morella faya* is reported here for the first time, although in non-English language documents, there are references to its medicinal use in the Canary Islands. This species requires in-depth investigation of its phytochemistry, toxicity and biological activity in vitro and in vivo in order to validate its efficacy as a medicinal plant.

The species *Clinopodium menthifolium* subsp. *ascendens* exhibits the highest UV and RFC values and is, therefore, the plant with the highest ethnomedicinal value for the Sete Cidades community.

Previously published studies on phytochemistry, pharmacology and toxicology are discussed and support the traditional uses of some of the reported plants, while in other cases, this does not happen. However, for several reported plants, no scientific studies were available to validate or rebuke their popular use. Therefore, it is necessary to research each species from the aforementioned perspectives in order to draw conclusions of their ethnopharmacological efficacy, relationships and safe uses in folk medicine practices.

It is noteworthy that some of the warnings from informants about the possibility of some species causing adverse effects on risk groups, such as children and pregnant women, are supported by scientific studies. 

Traditional knowledge is mainly in the custody of older members of the community. Informants indicate that fewer people use medicinal plants as folk remedies, a situation justified by new health systems and better access to urban centres. Nevertheless, plants are still used and play an important role in primary health care in the community of Sete Cidades. Due to the abundance of medicinal plants throughout the year, the conservation of the plants is minimal, and preferably, the fresh plant is used.

In the study area, a conservation/cultivation/trade program should be implemented as a contribution to preserving and adding value to this precious flora, while at the same time, a campaign is needed for the safeguard of traditional folk knowledge. In fact, through more scientific studies on local medicinal plants, the sustainable harvesting and commercialization of those plants could be achieved to improve the socio-economic situation of the population.

## Figures and Tables

**Table 1:** Medicinal plants used by the people of the Sete Cidades village.

Botanical name Family (Voucher)	Vernacular Name	Parts Used	Methods of Administration	Popular Uses and Treatment	UV ^a^	RFC ^b^	M/F ^c^
*Allium cepa* L. Amaryllidaceae (3620)	Cebola	Bulb peels	Inhalation and ingestion	Vapours from the infusion of the bulb peels mixed with *A. sativum* to treat colds and cough and the infusion to treat abdominal cramps. Decoction of the bulb peels mixed with leaves and bark of *E. umbellata*, *R. ulmifolius*, *P. cattleianum*, *A. sativum* and *M. sylvestris* to treat diarrhoea.	0.14	0.07	F
*Allium sativum* L. Amaryllidaceae (3621)	Alho	Bulb peels	Inhalation and ingestion	Vapours from the infusion of the bulb peels mixed with *A. cepa* to treat colds and cough and the infusion to treat abdominal cramps. Decoction of the bulb peels mixed with leaves and bark of *E. umbellata*, *R. ulmifolius*, *P. cattleianum*, *A. cepa* and *M. sylvestris* to treat diarrhoea.	0.11	0.07	F
*Aloysia citriodora* Palau Verbenaceae (3498)	Lúcia-lima	Leaves	Ingestion	Infusion to treat colds, nerves, tiredness, sputum and stomach pain. Decoction to treat nerves, hypertension, gallstones, menstrual pain and nasal congestion.	0.50	0.14	F
*Chelidonium majus* L. Papaveraceae (3492)	Celidónia	Stem	Dermal application	Latex or juice topically applied to treat scratches and wounds.	0.04	0.04	F
*Citrus limon* (L.) Osbeck Rutaceae (3490)	Limão	Peels	Ingestion	The juice of lemon, infusion and decoction of peels, simple or mixed with *M. x piperita*, coffee, ginger, honey and cinnamon, to treat colds, flu, arthritis and cirrhosis.	0.25	0.11	F
*Clinopodium menthifolium* subsp. *ascendens* (Jord.) Govaerts. Lamiaceae (3489)	Nêveda, neve, poejo miudinho, poejo	Whole plant and leaves	Ingestion, dermal application and inhalation	Decoction of the whole plant to treat bladder pain and urinary problems. The decoction of the leaves mixed with ginger (*Zingiber officinale*), honey and cinnamon (*Cinnamomum* spp.) stick to treat colds, gallbladder pain and flu. Infusion of leaves to treat abdominal cramps and stomach pain. Infusion and decoction of leaves to treat nerves, stomach pain and abdominal cramps. The leaves juice topically applied to treat mosquito bites and used as insect repellent. Smoking a cigarette made with the leaves to treat toothache.	0.71	0.43	M/F
*Dysphania ambrosioides* (L.) Mosyakin & Clemants Amaranthaceae (3508)	Usai-dela	Leaves and flower	Ingestion	Infusion and the juice of leaves and flowers to kill intestinal worms.	0.04	0.04	F
*Elaeagnus umbellata* Thunb. Elaeagnaceae (3504)	Bagueira	Leaves	Ingestion	Decoction of leaves mixed with leaves and bark of *M. sylvestris*, *R. ulmifolius*, *P. cattleianum*, *A. sativum* and *A. cepa* to treat diarrhoea.	0.04	0.04	F
*Equisetum telmateia* Ehrh. Equisetaceae (3509)	Rabo-de-asno	Leaves and stem	Ingestion	Infusion of leaves and stems to treat bladder pain and urinary problems.	0.04	0.04	F
*Eucalyptus globulus* Labill. Myrtaceae (3510)	Eucalipto	Leaves and flower	Inhalation, ingestion and dermal application	Infusion, decoction (and their vapours) of leaves and flowers to treat flu. The poultice of leaves mixed with oil, topically applied to help in rheumatism.	0.07	0.04	F
*Foeniculum vulgare* Mill. Apiaceae (3491)	Funcho	Seed, root, leaves and flower	Gargle and ingestion	Gargling with infusion of the seeds to treat cough and hoarseness. Decoction of seeds or roots to treat menstrual pain and flowers soup to treat liver problems. Decoction of the leaves or seeds to treat abdominal cramps, colds and stomach pain.	0.25	0.07	F
*Fragaria vesca* L. Rosaceae (3488)	Morangueiro	Leaves	Ingestion	Infusion of leaves to treat bladder pain and urinary problems.	0.04	0.04	F
*Hedera helix* L. Araliaceae (3503)	Hera	Leaves	Ingestion	Infusion of leaves to treat bladder pain and urinary problems.	0.04	0.04	F
*Malva sylvestris* L. Malvaceae (3501)	Malva	Leaves and whole plant	Dermal application, gargle and ingestion	Poultice of the leaves or the whole plant topically applied in wounds. Infusion and maceration of the leaves or whole plant topically applied to treat acne. Gargling with infusion of the leaves or whole plant to treat oral inflammation. Decoction of leaves mixed with leaves and bark of *E. umbellata*, *R. ulmifolius*, *P. cattleianum*, *A. sativum* and *A. cepa* to treat diarrhoea.	0.18	0.11	M/F
*Melissa officinalis* L. Lamiaceae (3505)	Cidreira	Leaves	Dermal application and ingestion	Decoction of leaves can be ingested or topically applied in a shower to treat nerves, insomnia and heart problems.	0.11	0.04	F
*Mentha spicata* L. Lamiaceae (3646)	Hortelã	Leaves	Ingestion	Decoction of leaves to treat indigestion and kill intestinal worms.	0.07	0.04	F
*Mentha x piperita* L.Lamiaceae (3506)	Hortelã-pimenta	Leaves	Inhalation, ingestion and gargle	Ingestion or gargle with the infusion or decoction of leaves to treat flu, stomach pain, pains of different aetiology, indigestion, and to kill intestinal worms. Vapours from the infusion to treat nasal congestion.	0.29	0.11	M/F
*Morella faya* (Aiton) Wilbur Myricaceae (3493)	Faia, Faia-da-terra; Faia-das-ilhas	Seed and leaves	Dermal application and ingestion	Poultice of burnt seeds mixed, or not, with olive oil and applied in wounds. Decoction of the leaves applied in the scalp to fortify the hair; and ingested to treat abdominal cramps and leg pain.	0.14	0.07	F
*Origanum majorana* L. Lamiaceae (3487)	Ourego	Leaves	Ingestion	Decoction of leaves to treat menstrual pain.	0.04	0.04	F
*Petroselinum crispum* (Mill.) Fuss Apiaceae (3499)	Salsa	Leaves	Ingestion	Decoction of leaves to treat stomach and gallbladder pains and to improve memory.	0.11	0.04	F
*Psidium cattleianum* Afzel. ex Sabine Myrtaceae (3507)	Gouveira-vermelha	Leaves	Ingestion	Decoction of leaves to treat stomach pain, and mixed with *M. sylvestris*, *R. ulmifolius*, *E. umbellata*, *A. sativum* and *A. cepa* to treat diarrhoea.	0.07	0.07	F
*Rosmarinus officinalis* L. Lamiaceae (3502)	Alecrim	Leaves and flower	Dermal application and ingestion	Shower with maceration of leaves and flowers to treat blood circulation problems. Decoction of leaves and/or flowers to treat stomach pain, abdominal cramps and headaches.	0.25	0.11	F
*Rubus ulmifolius* Schott Rosaceae(3496)	Silva	Shoot and bud	Ingestion	Infusion of shoot and bud to treat stomach pain. Decoction of shoot and bud mixed with *M. sylvestris*, *E. umbellata*, *P. cattleianum*, *A. sativum* and *A. cepa* to treat diarrhoea.	0.07	0.04	F
*Ruta chalepensis* L.Rutaceae(3497)	Arruda	Leaves	Ingestion	Infusion of leaves to treat pains of kidney, bladder, stomach and spine.	0.14	0.04	F
*Salvia officinalis* L. Lamiaceae (3500)	Salva	Leaves and flower	Ingestion	Decoction of leaves and flowers to treat oral inflammation and bronchitis. The infusion to treat stomach pain, nerves and cough.	0.18	0.07	F
*Sambucus nigra* L. Adoxaceae (3495)	Sabugueiro	Flower	Ingestion	Decoction of flowers to treat diarrhoea and fever.	0.07	0.04	F
*Taraxacum campylodes* G.E.Haglund Compositae (3593)	Dente-de-leão	Leaves	Ingestion	Salad and infusion of leaves to treat stomach pain, indigestion and used as laxative.	0.11	0.04	F
*Thymus vulgaris* L. Lamiaceae (3494)	Tomilho	Leaves	Ingestion	Infusion of leaves to treat stomach pain.	0.04	0.04	F
*Tilia × europaea* L. Malvaceae (3511)	Tília	Leaves and flower	Ingestion	Decoction of leaves and flowers to treat nerves.	0.04	0.04	F

^a^ UV = Use value; ^b^ RFC = Relative frequency of citation; ^c^ M/F = reported by Male (M) or Female (F).

**Table 2:** Comparative review of literature on the studied medicinal plants used in different regions across the Portugal.

Previous Study	Locality	Shared Species *
Novais et al. [22]	Arrábida Natural Park, Peninsula de Setúbal, southwestern Portugal	13
Neves et al. [23]	Montalegre and Chaves councils, Trás-os-Montes, northern Portugal	9
Carvalho and Morales [24]	Montesinho Natural Park, Trás-os-Montes, northeastern Portugal	5
Camejo-Rodrigues et al. [45]	Serra de São Mamede, Alentejo region, inner-central part of Portugal,	10
Rivera and Obón [79]	Madeira and Porto Santo Islands	6

* Shared species indicates how many plant species in the current study are common in comparison to previous studies on Portugal.

## Appendix A

**Table A1:** List of different recipes and posology used by people of the Sete Cidades community in São Miguel island.

Plant Name	Preparation Mode	Posology	Adverse Effects
*Allium cepa* L.	For the infusion, fresh and dried peels can be used, and for the decoction, fresh peels. Inhale vapors of the infusion of the bulb peels mixed with *A. sativum* for 10 minutes. When infusing, don’t let it boil. To prepare the decoction, mix the bulb peels with leaves and bark of *E. umbellata*, *R. ulmifolius*, *P. cattleianum*, *A. sativum* and *M. sylvestris*, plenty of each ingredient to ¾ L of water. At the end, remove the *M. sylvestris*. Peels can be conserved dried.	Drink the infusion and inhale its vapors before going to bed and when waking up. Drink the decoction three times a day: in the morning, lunch time and night.	Do not give to children younger than 5 years old
*Allium sativum* L.	For the infusion and the decoction fresh and dried peels can be used. Inhale vapors of the infusion of the bulb peels mixed with *A. cepa* for 10 minutes. When infusing, don’t let it boil. To prepare the decoction, mix the bulb peels with leaves and bark of *E. umbellata*, *R. ulmifolius*, *P. cattleianum*, *A. cepa* and *M. sylvestris*, plenty of each ingredient to ¾ L of water. At the end, remove the *M. sylvestris*. Peels can be conserved dried.	Drink the infusion and inhale its vapors before going to bed and when waking up. Drink the decoction three times a day: in the morning, lunch time and night.	Do not give to children younger than 5 years old
*Aloysia citriodora* Palau	For the infusion, use fresh leaves, and for the decoction fresh and dried leaves can be used. To treat colds, infuse 15 leaves per liter of water. To prepare the infusion and the decoction for other medicinal uses, infuse 3 to 4 soup spoons of leaves per liter of water. Leaves can be conserved dried.	To treat colds, drink the infusion every day. Drink the other infusion or decoction for other medicinal uses throughout the day, without drinking it many days in a row.	Infusion made for colds should not be given to children younger than 5 years old
*Chelidonium majus* L.	Use fresh stem. Extract the juice or latex and apply in the wound until it gets dark. Stem is not conserved.	Apply only once directly in the wound until it gets dark.	No
*Clinopodium menthifolium* subsp. *ascendens* (Jord.) Govaerts	Use leaves or whole plant fresh or dried. To prepare the decoction: to use in colds and flu, gallbladder pain, mix dried or fresh leaves with 3 coffee spoons of ginger, 1 spoon of honey and 1 cinnamon stick; to use in urinary problems and bladder pain, decoct 3 to 4 leaves per liter of water, and mix with other plants if desired. To prepare the infusion or decoction to use in stomach pain or abdominal cramps, infuse or decoct 15 g/L of water. Use fresh leaves to prepare juice. Whole plant and leaves can be conserved dried and closed away from direct sun light to prepare a cigarette.	Decoction for colds and flu can be drinked as desired. Drink infusion or decoction for stomach pain and abdominal cramps only when in pain. Drink 1.5 L of decoction for urinary problems or bladder pain.	No
*Citrus limon* (L.) Osbeck	Use fresh peels. To prepare the infusion and the decoction use lemon peel according to taste. Juice can be diluted. They can be mixed with only *M. x piperita* or adding more 1 coffee spoon of ginger, 1 spoon of honey and 1 spoon of cinnamon. Peels can be conserved in the fridge or frozen.	The infusion, decoction and juice (diluted or not) should be drinked whenever feeling sick or before going to bed.	No
*Dysphania ambrosioides* (L.) Mosyakin & Clemants	To prepare the infusion use fresh leaves and flowers according to taste. Leaves and flowers are not conserved.	As desired.	No
*Elaeagnus umbellata* Thunb.	Use fresh leaves. To prepare the decoction, mix the bulb peels with leaves and bark of *M. sylvestris*, *R. ulmifolius*, *P. cattleianum*, *A. sativum* and *A. cepa*, plenty of each ingredient to ¾ L of water. At the end, remove the malvas. The leaves are not conserved.	Drink the decoction three times a day: in the morning, lunch time and night.	Do not give to children younger than 5 years old
*Equisetum telmateia* Ehrh.	Use fresh leaves and stem. Infuse 15 g/L of water. Leaves are not conserved.	Prepare and drink only when in pain or urinary problems.	No
*Eucalyptus globulus* Labill.	Leaves and flowers can be used fresh or dried. To prepare the infusion or decoction use 25 to 30 g/L of water. Leaves and flowers can be conserved dried.	Drink 2 to 3 teacups a day.	No
*Foeniculum vulgare* Mill.	Use fresh flowers and roots, and dried leaves. Seeds can be used dried and fresh. To prepare the infusion to gargle, use 1 teaspoon of seeds for one glass of water. To prepare decoction against menstrual pain, use 20 g of fresh seeds or 30 g of fresh roots per litre of water; for abdominal cramps, colds or stomach pain, use 20 g of dried seeds or 20 g of dried leaves per litre of water. Prepare a flower soup according to taste, to treat liver problems. The infusion, decoction or soup using fresh ingredients cannot be conserved. Leaves and seeds can be conserved dried in a dry place.	Gargle with infusion when in pain. Decoction used for menstrual pain should be ingested three days before menstruation. Flower soup should be consumed continuously. Decoction used in abdominal cramps, colds or stomach pain should be given to babies and alternated with breast milk.	No
*Fragaria vesca* L.	Use fresh leaves only. Infuse 15 g/L of water. Leaves are not conserved.	Prepare and drink only when in pain or urinary problems.	No
*Hedera helix* L.	Use fresh leaves only. Infuse 15 g/L of water. Leaves are not conserved.	Prepare and drink only when in pain or urinary problems.	No
*Malva sylvestris* L.	Use fresh leaves and whole plant. To prepare the poultice boil 30 to 40 leaves per litre of water. To prepare the infusion and maceration, use 20 leaves per litre of water. To gargle, use infusion. To prepare the decoction, mix the leaves with leaves and bark of *E. umbellata*, *R. ulmifolius*, *P. cattleianum*, *A. sativum* and *A. cepa*, plenty of each ingredient to ¾ L of water. The plant is not conserved.	Apply the poultice until the wound heals. Wash the face with the infusion every day, and gargle with it until fully recovered. Drink the decoction 3 times a day: in the morning, at lunch time and night.	Do not give to children younger than 5 years old
*Melissa officinalis* L.	Prepare the decoction using fresh leaves to taste. Leaves are not conserved.	Use the decoction in the soaking bath before going to bed. Drink 1 to 2 times a day.	Do not give to children younger than 5 years old
*Mentha spicata* L.	Use fresh leaves. To prepare the decoction, use 10 to 30 g/L of water. Leaves are not conserved	Drink at lunch and dinner.	No
*Mentha × piperita* L.	Use fresh leaves. To prepare the infusion or decoction to drink or gargle, use a couple of leaves for ½ L of water. Leaves are not conserved. The drink can be conserved in the fridge.	Drink the infusion or decoction, or gargle with it, during the day as desired, at room temperature. Inhale the vapors from the infusion for 10 minutes.	Do not give to children younger than 5 years old
*Morella faya* (Aiton) Wilbur	Use dry seeds and fresh leaves. To prepare a poultice, burn the dried seeds, and mix them if desired with olive oil, to create a past to apply topically. Prepare the decoction using fresh leaves according to taste. Seeds are conserved dried.	Apply the poultice once a day in the wound until it closes. Rub the scalp during the bath with the decoction. Drink the decoction when having abdominal cramps or leg pain.	Do not give to children younger than 5 years old
*Origanum majorana* L.	Prepare the decoction using fresh leaves according to taste. Leaves are not conserved.	As desired.	No
*Petroselinum crispum* (Mill.) Fuss	Use fresh leaves. To prepare decoction, use leaves of 1 to 2 twigs per coffee pot. Leaves are not conserved.	As desired.	Do not give to children younger than 5 years old
*Psidium cattleianum* Afzel. ex Sabine	Use fresh or dried leaves. To prepare the decoction: to use in stomach pain, use 3 to 4 fresh or dried leaves per litre of water; to use in diarrhea, mix the fresh leaves with *M. sylvestris*, *R. ulmifolius*, *E. umbellata*, *A. sativum* and *A. cepa*, plenty of each ingredient. At the end, remove the malvas. Leaves are conserved dried.	Drink 1.5 L a day of decoction for stomach pain until feeling better. Drink the decoction for diarrhea 3 times a day: morning, lunch time and night.	Do not give to children younger than 5 years old
*Rosmarinus officinalis* L.	Use fresh leaves and flowers. Prepare the maceration according to taste. To prepare the decoction use 20 to 30 g of fresh leaves and/or flowers per litre of water. Leaves and flowers can be conserved dried.	Use the maceration in the soaking bath but only in the morning and never at night-time as it disturbs the sleep. Drink the decoction when in pain.	No
*Rubus ulmifolius* Schott	Use fresh or dried shoots and buds. Infuse 30 g/L of water of fresh or dried shoots and buds. To prepare the decoction, use fresh shoots and buds mixed with *M. sylvestris*, *E. umbellata*, *P. cattleianum*, *A. sativum* and *A. cepa*, plenty of each ingredient to ¾ L of water. At the end remove the malvas. Shoots and buds are conserved dried.	Both the infusion and the decoction should be drunk 3 times a day: morning, at lunch time and night.	Do not give to children younger than 5 years old
*Ruta chalepensis* L.	Use fresh leaves. Infuse up to a maximum of 20 g/L of water of fresh leaves. Leaves are not conserved.	Drink throughout the day.	Infusing more than 20g of leaves per liter gets toxic. Do not give to children younger than 5 years old
*Salvia officinalis* L.	Use fresh or dried leaves and flowers. Decoct 30 g of fresh or dried leaves and flowers per litre of water. The infusion is prepared according to taste. Leaves are conserved dried.	As desired.	No
*Sambucus nigra* L.	Use fresh flowers. Decoct 10 to 15 flowers per litre of water. Flowers are not conserved.	Drink 3 cups a day.	Do not give to children younger than 5 years old
*Taraxacum campylodes* G.E.Haglund	Use fresh or dried leaves. The infusion is prepared according to taste. Use fresh leaves for salads in meals. Leaves are conserved dried.	Drink and consume continuously.	No
*Thymus vulgaris* L.	Use fresh leaves. Infuse. Leaves are not conserved.	As desired.	No
*Tilia × europaea* L.	Use fresh or dried leaves and flowers. Decoct 30 g/L of water of fresh or dried leaves and/or flowers. The infusion is prepared according to taste. Leaves and flowers are conserved dried.	As desired.	No

**Table A2:** Presence and absence of the studied species in the context of previous literature. A [79]; B [45]; C [22]; D [23]; E [24].

Plant Species	A	B	C	D	E	Frequency	Absolute
*Allium cepa* L.	0	1	1	0	0	2	1
*Allium sativum* L.	0	0	0	0	0	0	0
*Aloysia citriodora* Palau	0	0	0	0	0	0	0
*Chelidonium majus* L.	0	0	1	1	1	3	1
*Clinopodium menthifolium* subsp. *ascendens* (Jord.) Govaerts	0	0	0	0	0	0	0
*Dysphania ambrosioides* (L.) Mosyakin & Clemants	0	0	0	0	0	0	0
*Citrus limon* (L.) Osbeck	0	1	1	0	0	2	1
*Elaeagnus umbellata* Thunb.	0	0	0	0	0	0	0
*Equisetum telmateia* Ehrh.	0	0	1	0	0	1	1
*Eucalyptus globulus* Labill.	0	0	1	0	0	1	1
*Foeniculum vulgare* Mill.	1	1	1	1	1	5	1
*Fragaria vesca* L.	0	1	0	0	1	2	1
*Hedera helix* L.	0	0	0	1	1	2	1
*Malva sylvestris* L.	0	1	1	0	0	2	1
*Melissa officinalis* L.	0	1	1	1	0	3	1
*Mentha spicata* L.	0	0	0	0	0	0	0
*Mentha × piperita* L.	0	1	1	1	0	3	1
*Morella faya* (Aiton) Wilbur	0	0	0	0	0	0	0
*Origanum majorana* L.	0	0	0	0	0	0	0
*Petroselinum crispum* (Mill.) Fuss	0	0	1	0	0	1	1
*Psidium cattleianum* Afzel. ex Sabine	1	0	0	0	0	1	1
*Rosmarinus officinalis* L.	0	1	1	1	0	3	1
*Rubus ulmifolius* Schott	0	1	1	0	0	2	1
*Ruta chalepensis* L.	1	0	1	0	0	2	1
*Salvia officinalis* L.	1	0	0	1	0	2	1
*Sambucus nigra* L.	0	1	0	1	1	3	1
*Taraxacum campylodes* G.E.Haglund	0	0	0	0	0	0	0
*Thymus vulgaris* L.	1	0	0	0	0	1	1
*Tilia × europaea* L.	0	0	0	0	0	0	0
**Total**	**6**	**10**	**13**	**9**	**5**		**19**
